# Entry of Bluetongue Virus Capsid Requires the Late Endosome-specific Lipid Lysobisphosphatidic Acid*

**DOI:** 10.1074/jbc.M115.700856

**Published:** 2016-04-01

**Authors:** Avnish Patel, Bjorn-Patrick Mohl, Polly Roy

**Affiliations:** From the Department of Pathogen Molecular Biology, Faculty of Infectious and Tropical Diseases, London School of Hygiene and Tropical Medicine, Keppel Street, London WC1E 7HT, United Kingdom

**Keywords:** endosome, lysophospholipid, membrane, virus, virus entry, bluetongue virus, lipid vesicle, lipid-protein interaction, lysobisphosphatidic acid

## Abstract

The entry of viruses into host cells is one of the key processes of infection. The mechanisms of cellular entry for enveloped virus have been well studied. The fusion proteins as well as the facilitating cellular lipid factors involved in the viral fusion entry process have been well characterized. The process of non-enveloped virus cell entry, in comparison, remains poorly defined, particularly for large complex capsid viruses of the family Reoviridae, which comprises a range of mammalian pathogens. These viruses enter cells without the aid of a limiting membrane and thus cannot fuse with host cell membranes to enter cells. Instead, these viruses are believed to penetrate membranes of the host cell during endocytosis. However, the molecular mechanism of this process is largely undefined. Here we show, utilizing an *in vitro* liposome penetration assay and cell biology, that bluetongue virus (BTV), an archetypal member of the Reoviridae, utilizes the late endosome-specific lipid lysobisphosphatidic acid for productive membrane penetration and viral entry. Further, we provide preliminary evidence that lipid lysobisphosphatidic acid facilitates pore expansion during membrane penetration, suggesting a mechanism for lipid factor requirement of BTV. This finding indicates that despite the lack of a membrane envelope, the entry process of BTV is similar in specific lipid requirements to enveloped viruses that enter cells through the late endosome. These results are the first, to our knowledge, to demonstrate that a large non-enveloped virus of the Reoviridae has specific lipid requirements for membrane penetration and host cell entry.

## Introduction

Cell entry of viruses is a key stage that initiates infection. During this process, viruses must breach the membrane barrier that encompasses cellular contents (1–3). The site of virus membrane penetration is specific to each virus; however, the majority of viruses enter the cytosol via the endocytic compartments of host cells (4, 5). In this environment, acidic pH triggers virus fusion proteins that mediate virus entry into the host cell cytosol. This process has been particularly well characterized in enveloped viruses that enter the host cytosol by fusing their own limiting membrane with that of the host cell, thereby releasing viral contents to initiate the infection cycle (6–8). Non-enveloped viruses do not possess a limiting membrane and as such must traverse the membrane barrier of the cell by an alternative mechanism. This process is well understood for small non-enveloped viruses, such as members of the Picornaviridae, in which evidence suggests that small amphipathic peptides insert and form a discrete pore in the host membrane through which viral contents are extruded (9–13). For larger non-enveloped viruses, such as those of the Reoviridae, the mechanism of host cell membrane disruption remains poorly defined. The Reoviridae comprises a large family of non-enveloped viruses whose capsids are complex and encapsidate a segmented double-stranded RNA genome (14). These viruses initiate host cell infection by delivering a large core particle into the host cell cytosol; however, the mechanism by which these large particles traverse the cellular membrane barrier remains poorly understood. Several members of the Reoviridae have been shown to enter the cell cytosol via the early-late endocytic compartments. Although some insights have been gained into the process of penetration by mammalian Reovirus and Rotavirus, these studies have been conducted with membranes that do not accurately represent the membrane environment present during trafficking through the cellular endocytic compartments (15–18). BTV^2^ is an archetypal member of the *Orbivirus* genus of the family Reoviridae. BTV consists of 27 serotypes (19) and is an agriculturally significant arbovirus that causes a hemorrhagic disease in undulates, predominantly in sheep (20, 21); however, recent outbreaks of BTV serotype 8 have also shown pathogenicity in domestic cattle herds (22, 23). The virus consists of three concentric layers of protein (24, 25) with the innermost layers of VP3 and VP7 delimiting the structure of the core particle (26–28) that enters the host cytosol (29). The outer layer of the virus capsid is composed of VP2 and VP5 proteins (30) that facilitate virus entry and delivery of the core particle into the host cell cytosol (31). VP2 has been shown to act as a receptor-binding protein, which binds sialic acid (32, 33) and facilitates clathrin-mediated endocytosis of the viral particle that is trafficked into the endosomal compartments of the cell (34). VP5 acts as an acid-dependent membrane penetration protein that penetrates the host cell membrane (35) and delivers the core particle into the host cytosol, wherein transcription of the viral genome commences (36). How this protein penetrates cellular membranes and which membrane factors facilitate this process are poorly characterized.

Here, using BTV as a model system, we investigate the membrane composition involved in VP5 membrane penetration. Using an *in vitro* liposome penetration assay, we demonstrate that VP5 penetrates liposomes of a late endosome (LE), but not early endosome (EE), membrane composition and that this is due to the late endosome-specific lipid factor 2,2′-dioleoyl lysobisphosphatidic acid (LBPA). We demonstrate that this VP5-dependent penetration process is probably due to a combination of anionic charge and fluidic properties of LBPA. Further, we show that VP5 forms pores of a discrete size and that LBPA may allow VP5 membrane pore expansion in a concentration-dependent manner. We corroborate these *in vitro* findings pharmacologically in *in vivo* virus infection, which suggests that BTV enters via the LE compartment because its membrane composition allows efficient pore formation for core delivery to the host cell cytosol. These findings demonstrate a specific reliance of a non-enveloped virus on a host lipid factor for cell entry, due to its biophysical properties. This relationship may hold true for other non-enveloped viruses that deliver large cargos into the host cytosol, presenting a novel therapeutic avenue for infection prophylaxis of these virus types.

## Experimental Procedures

### 

#### 

##### Cell Lines and Virus Stocks

BSR, HeLa, and PT cells were maintained as described previously (37, 38). *Spodoptera frugiperda* (*Sf9*) cells were grown in InsectExpress (Lonza) medium supplemented with 2% (v/v) fetal calf serum and incubated either in suspension or in monolayer cultures at 28 °C. Recombinant *Autographa californica* nuclear polyhedrosis viruses were produced by co-transfecting pTriExHMBPVP5 WT or mutant plasmid and Bacmid:KO *orf*1629 (39). Expression cultures were maintained as described previously (35).

##### Antibodies, Reagents, and Plasmids

Polyclonal antibodies were used for detection of viral proteins NS1 (produced in mouse) and NS2 and VP5 (produced in guinea pig); these were produced in house. For detection of LBPA, a mouse monoclonal antibody, anti-LBPA antibody (6C4), in cell supernatant (Z-SLBPA) was purchased from Echelon Biosciences Inc. for the detection of LBPA. A rabbit polyclonal antibody, anti-GAPDH (ab9485), and a polyclonal anti-Lamp1 rabbit antibody (ab24170-100) were purchased from Abcam. For antibody uptake blocking assays, a purified version of the monoclonal anti-LBPA antibody (P-SLBPA) (Echlelon Biosciences Inc.) and a monoclonal anti-FLAG® M2 mouse antibody (Sigma-Aldrich) were utilized. Hoechst 33342 was purchased from Life Technologies, Inc. Alexa 488- and 594-conjugated secondary antibodies were purchased from Life Technologies and Abcam, respectively. Alkaline phosphatase-conjugated goat anti-mouse, anti-rabbit, and anti-guinea pig polyclonal antibodies were obtained from Sigma-Aldrich. Paraformaldehyde, BSA, Triton X-100, alkaline phosphatase liquid ELISA substrate, DMSO, and U18886A inhibitors were purchased from Sigma-Aldrich. Calcein and fluorescent dextrans of 4, 10, and 20 kDa were purchased from Sigma-Aldrich. Lipids phosphatidylcholine (PC), phosphatidylethanolamine, sphingomyelin, phosphatidylinositol, phosphatidylserine (PS), LBPA, and Avanti® Mini-Extruder were purchased from Avanti Polar lipids. 0.1-μm Nuclepore Track-Etched Membranes polycarbonate membrane filters were purchased from Whatman. The pTriExHMBP-TEV-VP5 transfer vector was constructed by cloning the coding region of BTV-1 VP5 into a BamHI site of a premade (in house) empty pTriExHMBP-TEV vector in which cloning into a BamHI site places the gene of interest in-frame with and upstream of the HMBP-TEV cassette for which the GS of the ENLYFQGS TEV site represents the in-frame translation of the BamHI restriction site.

##### Recombinant VP5 Expression and Purification

BTV-1 VP5 (accession number ACR58462) proteins were expressed using a recombinant baculovirus system in *Sf9* cells. Recombinant VP5 was expressed as an N-terminally tagged His_6_-MBP fusion protein with a glycine-serine linker and a TEV cleavage site. Expression cultures were harvested 50 h postinfection, and cells were lysed by Dounce homogenization in lysis buffer (20 mm Tris, 150 mm NaCl, 20 mm imidazole, 1% Triton X-100, pH 8.5) supplemented with EDTA-free protease inhibitor mixture (Sigma-Aldrich), and lysate was clarified by centrifugation. Soluble lysate was purified using an Äkta Explorer FPLC unit (GE Healthcare), first utilizing immobilized metal affinity chromatography with a 5-ml HisTrap HP column (GE Healthcare) and, second, affinity chromatography using a 1-ml MBPTrap HP column (GE Healthcare). Eluted proteins were concentrated to 200 μl and cleaved with 20 μg of TEV protease (Sigma-Aldrich) overnight at 4 °C. Cleaved tags and proteases were removed using immobilized metal affinity chromatography. Typically, 0.5 mg of pure WT VP5 was produced per 200 ml of culture. Mutant proteins were produced by site-directed mutagenesis of WT expression constructs. Primers were phosphorylated using polynucleotide kinase (New England Biolabs), and forward primers Mutant1 (5′-GATGGGT**CT**AGTCATAC**T**GTCCTTAAACCGATTTGGCAAAAG-3′), Mutant2 (5′-GATGGGT**CT**AGTCATAC**T**GTCCTTAAACC**T**ATTTGGC**CT**A**CT**GGTAGGCAACGCGTTAACTTCTAATAC-3′), and Mutant3 (5′-GATGGGTAAAGTCATACGGTCCTTAAACC**T**ATTTGGC**CT**A**CT**GGTAGGCAACGCGTTAACTTCTAATAC-3′) were utilized in conjunction with the reverse primer mut rev (5′-TTCGCTAAGGGCGCACTTTTTAAC-3′) to amplify WT BTV-1 VP5 (accession number ACR58462) pUC19 T7 plasmid (boldface lettering indicates mutation sites). The products were DpnI (New England Biolabs)-digested and then ligated and transformed. Mutations were confirmed by Sanger sequencing and used as they were for reverse genetics or mutant ORFs cloned into the pTriExHMBP-TEV vector for protein expression.

##### Liposome Production

A total of 5 mg of lipids (Avanti Polar Lipids) with a composition of early or late endosomal membranes (see Table 1) in chloroform were dried under vacuum to give a solvent-free film. This was either hydrated in 1 ml of Calcein (Sigma-Aldrich) buffer (50 mm calcein, 100 mm NaCl, 2.7 mm KCl, 10 mm Na_2_HPO_4_, 1.8 mm KH_2_PO_4_, pH 7.5) or, for fluorescent dextrans (Sigma-Aldrich), PBS buffer (137 mm NaCl, 2.7 mm KCl, 10 mm Na_2_HPO_4_ 1.8 mm KH_2_PO_4_ pH 7.5) plus dextran at a concentration of 200 mg ml^−1^. Mixtures were subsequently freeze-thawed three times with brief vortexing after each thaw. The lipid suspensions were then extruded 11 times through a 0.1-μm polycarbonate membrane filter (Whatman) using a Mini Extruder (Avanti Polar Lipids). Unencapsulated calcein and fluorescent dextran were removed from the extruded suspension by size exclusion chromatography using Sephadex G-75 resin (Sigma-Aldrich), whereas PBS, pH 7.5 (liposome buffer) was used as the aqueous phase buffer. Liposomes were stored at 4 °C and used within 1 week of creation.

##### Pore Formation Assay

Purified WT and mutant VP5 proteins were reconstituted in PBS, pH 7.5, containing 1 μg/ml anti-gp64 monoclonal antibody B12D5 (liposome buffer). Calcein or dextran-loaded liposomes and VP5 protein were mixed in a flat black 96-well plate (Griener) and incubated at room temperature for 10 min at a final concentration of 0.1 mg/ml VP5 and 0.1 mg/ml total lipid for liposomes. Mixtures were then acidified to the stated pH levels using a predetermined titration of 0.1 n HCl and incubated at 37 °C for 30 min. Fluorescence was then measured with a SpectraMax® M5 plate reader (Molecular Devices) using top read mode with an excitation/emission of 485/535 nm. All liposome data were normalized to complete calcein release control using Triton X-100 buffer and buffer alone, which included the gp64 antibody. Normalized percentage calcein or dextran release was calculated using the formula, *R*% = (*R* − *R*_0_)/(*R*_100_ − *R*_0_), where *R*% is percentage release, *R* is the measured fluorescence, *R*_0_ is the measured fluorescence of liposomes acidified with buffer alone, and *R*_100_ is the fluorescence of liposomes with buffer containing 0.1% Triton X-100. Experiments were performed in triplicate.

##### Reverse Genetics

VP5 mutants were created by site-directed mutagenesis of WT BTV-1 VP5 (accession number ACR58462) pUC19 T7 plasmid. Reverse genetics was performed as described previously (40) and repeated three times. In all cases, only WT virus was recovered.

##### Cholesterol Transport Inhibitor U18666A Treatment of Viral Infection

1 × 10^4^ HeLa or PT cells/well of a 96-well culture dish were incubated for 2 h with medium containing 30 μm U18666A, a non-toxic concentration, by trypan blue staining. Subsequently, wells were washed with serum-free drugged or mock-treated (drug carrier buffer) medium. Cells were synchronously infected with BTV-1 in serum-free drugged or mock-treated medium at an MOI of 0.1, 1, or 5. After virus adsorption, cells were washed twice and then incubated with drug- or mock-treated medium for a further 3 h at 35 °C, after which medium was replaced with drug-free medium. Plates were further incubated at 35 °C for 9 h, after which wells were washed and fixed with 4% formaldehyde in PBS at room temperature for 15 min.

##### Antibody Endocytosis-blocking Assay

1 × 10^4^ HeLa cells/well of a 96-well culture dish were incubated with medium containing 50 μg ml^−1^ of an anti-LBPA mouse antibody or isotype-matched control anti-FLAG antibody (Sigma-Aldrich) for 18 h. Cells were then washed and synchronously infected with BTV-1 at an MOI of 1. After virus adsorption, infected cells were washed and incubated in fresh medium at 35 °C for 12 h, after which cells were fixed as for drug-treated cells.

##### Western Blotting Analysis

SDS-polyacrylamide gels were transferred via a semidry blotter to PVDF transfer membranes and blocked for 4 h with TBS-T containing 10% (w/v) milk powder. Primary antibodies for the detection of NS1 and NS2 were rabbit anti-NS1 serum and guinea pig anti-NS2 serum, and commercial rabbit anti-GAPDH (Abcam, ab9485) was used for GAPDH. Blot images were imported into ImageJ software for densitometry analysis. The background intensity was subtracted from the images. Bands were then selected by drawing a tight boundary around them. Intensities of the selected bands were then exported into an Excel format. NS1 and NS2 values were normalized to the corresponding GAPDH values to generate NS1 or NS2/GAPDH ratios for each sample for further statistical analyses.

##### Immunoperoxidase Assay

Fixed cells in a 96-well format were blocked and permeabilized by incubation with blocking buffer (PBS, 1% (w/v) BSA, 0.1% Triton X-100) for 1 h. A mouse primary antibody (produced in this laboratory) to the viral non-structural protein antigen NS1 was then bound (1:300 dilution) by incubating wells for 1 h at room temperature. Subsequently, cells were washed three times, and a secondary alkaline phosphatase conjugate anti-mouse IgG secondary antibody (1:500 dilution) (Sigma-Aldrich) was bound for 1 h at room temperature. Cells were subsequently washed, followed by the addition of alkaline phosphatase yellow (*p*-nitrophenyl phosphate) liquid substrate (Sigma-Aldrich) to each well. Signal was developed at room temperature for 20 min, after which the reaction was stopped by the addition of NaOH. Plates were then read at an absorbance of 405 nm with a SpectraMax® M5 plate reader (Molecular Devices) using top read mode. Averaged signal from uninfected and cells was subtracted from the data, and percentage infectivity was calculated by normalizing to infected mock-treated cells.

##### Confocal Microscopy

Confocal microscopy was performed by a method similar to that of Du *et al.* (38). Briefly 5 × 10^4^ HeLa cells were synchronously infected with BTV-1 at an MOI of 10. Unbound virus was subsequently washed off, and coverslips were fixed at a time point either 0, 15, 20, or 45 min after infection. Coverslips were then processed for imaging. Slides were imaged using a Zeiss LSM510 confocal microscope using oil immersion with a ×63 objective at room temperature. Images were processed using a Zeiss image browser.

##### Virus Titration

Supernatants from BTV-infected cells were collected after 24 h, and relative virus titers were determined by plaque assays on BSR cells. Briefly, cells were seeded in 12-well plates and incubated for 45 min with diluted virus in 100 μl of serum-free DMEM. After removal of the inoculums, cells were washed once and overlaid with 1 ml of 0.6% Avicel-containing overlay medium containing 2% fetal bovine serum and antibiotics. Plaques were visualized after an incubation period of 2–3 days at 35 °C by staining with crystal violet for several hours.

##### Statistical Analysis

*p* values were calculated by unpaired *t* test using GraphPad® Prism 6 software.

## Results

### 

#### 

##### VP5 Penetrates Late Endosome-mimicking Liposomes

**FIGURE 1.**
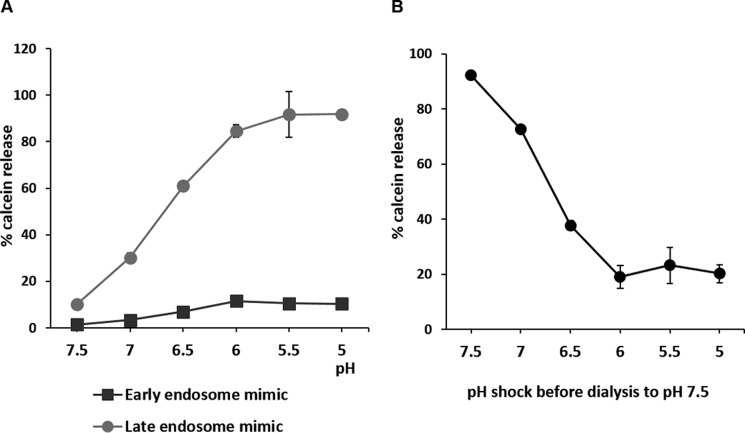
**The pH dependence of VP5-induced calcein release from 0.1-μm vesicles mimicking early and late endosome membranes.** *A*, purified VP5 was mixed with either early or late endosome-mimicking liposomes and acidified to a range of pH as indicated. Leakage of calcein from liposomes was assayed, and the results were normalized to liposomes treated or untreated with 0.1% Triton X-100. *B*, VP5 protein was acidified to pH 5.0–7.0, as indicated, and subsequently dialyzed back to pH 7.5. A pore formation assay was then performed with acidification at pH 5.5. Results were normalized as in *A*. Results show three independent repeats, and *error bars* represent S.D.

Recent cell biology data indicate that BTV serotype 1 traffics from early to late endosomal compartments, with VP2 and VP5 found both in the EE compartment and VP5 alone in the LE compartment (38). Although this indicates the localization of these proteins during entry, the site at which BTV core enters into the cytosol has not clearly been demonstrated. The environment of EE and LE compartments has been shown to vary significantly, with each compartment displaying a unique lipid composition of membranes (41, 42), with an increased acidity occurring during trafficking toward the lysosome (43–45). Using an *in vitro* liposome penetration assay, it was investigated which membrane composition of EE or LE and at which pH VP5 mediates membrane pore formation. Liposomes were produced that approximated the composition of EE or LE endosomal membranes defined by Kobayashi *et al.* in 1999 (48) (Table 1). Baculovirus-expressed purified recombinant VP5, as described under “Experimental Procedures,” was utilized in a liposome penetration assay with liposomes mimicking EE or LE. Recombinant VP5 was shown to substantially penetrate liposomes of an LE in a pH-dependent manner with ∼81% (*p* < 0.001) penetration occurring between pH 7.5 and 5. In addition, the most efficient membrane penetration occurred at a late endosomal pH 5–5.5 for LE liposomes (Fig. 1*A*). A small (∼9%) but significant (*p* < 0.01) penetration of liposomes mimicking EE was observed between pH 7.5 and pH 5. We further corroborated these findings by determining the pH at which the conformational change of VP5 occurred. VP5 was pre-acidified to the pH values 7.5–5 (Fig. 1*B*) and then re-equilibrated to pH 7.5 prior to use in a liposome assay by acidification to pH 5. The results demonstrated that the conformational change of VP5 is irreversible and occurs substantially at pH 6.5, with maximal conformational change occurring at a pH of the LE (Fig. 1*B*). Together, these data strongly suggest that VP5 is activated at a pH of early to late endosomal transition and can actively penetrate LE membranes.

**TABLE 1:** **Lipid compositions of EE- and LE-mimicking liposomes** PE, phosphatidylethanolamine; SM, sphingomyelin; PI, phosphatidylinositol. Modified from Ref. 48.

	EE	LE
	%	%
PC	50	50
PE	23	20
SM	9	3
PS	9	4
PI	8	8
LBPA	1	15

##### Lysobisphosphatidic Acid Is Required for VP5 Late Endosome-mimicking Liposome Penetration

**FIGURE 2.**
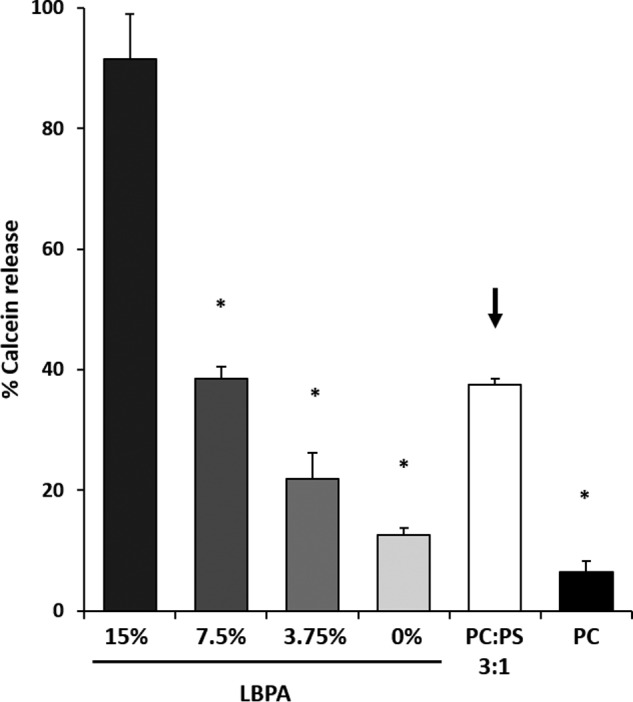
**The late endosome lipid LBPA facilitates VP5 penetration.** Late endosome-mimicking liposomes containing a decreasing amount of LBPA (swapped for PC) were tested in a pore formation assay with purified VP5 at pH 5.5. Significant results are indicated (*, *p* < 0.01 compared with LE 15% LBPA). Liposomes with a simplified high anionic (PC/PS, 3:1) and non-anionic composition (PC alone) were also tested (*, *p* < 0.01 compared with 1:3 PC/PS). *Arrow*, penetration recovery by including anionic PS with PC in the liposome membrane composition. Results from three independent repeats were normalized to liposomes treated or untreated with 0.1% Triton X-100. *Error bars*, S.D.

Considering the finding that VP5 pore formation occurs in LE membrane-mimicking liposomes, we subsequently investigated the lipid properties of LE membranes that allow VP5 penetration function. The lipid composition of LE membranes is significantly enriched in LBPA (Table 1) compared with EE. We hypothesized that this major difference in lipid composition could be the enabling factor in VP5 membrane penetration. To investigate this possibility, LE-mimicking liposomes were tested in which the LBPA content was decreased by substitution with the zwitterionic lipid PC, which constitutes the major lipid of LE membranes (Table 1). Results indicated a direct dependence of VP5 membrane penetration with LBPA content (Fig. 2), suggesting a role of this lipid in enabling VP5 membrane penetration. Further, we investigated the physical properties of this lipid that could allow membrane penetration. LBPA is anionic, which may be a contributing factor to allow VP5 membrane pore formation. To test this, we created liposomes of a simpler membrane composition. PS is another anionic lipid that is found in the inner leaflet of the cytosolic membrane and to some extent in EE and less so LE membranes (46). Liposomes containing PC alone or PS/PC (1:3) were tested to investigate whether the artificially high 33% anionic charge of PS in these liposomes could also facilitate membrane penetration by VP5. Results suggested that in a simple membrane composition, PS was able to partly facilitate membrane penetration by VP5. However, this was not as efficient as LE membranes (Fig. 2, *arrow*), suggesting an effect of anionic charge in enabling VP5 penetration activity.

**FIGURE 3.**
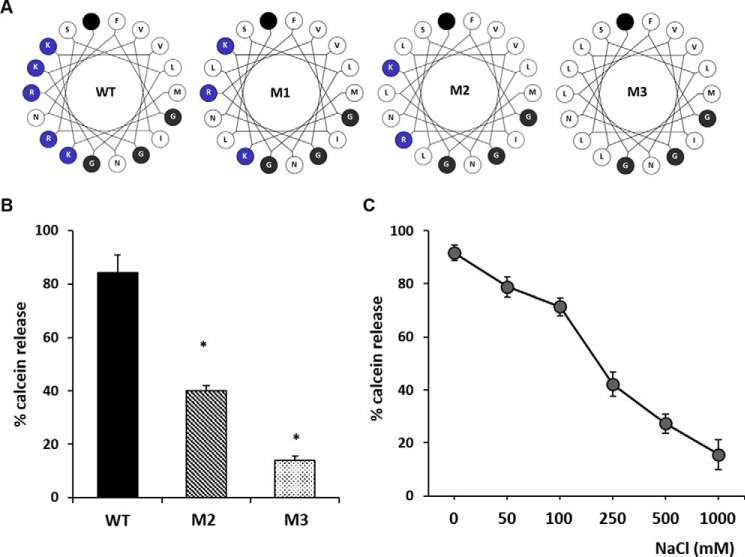
**Positive charged residues in the N-terminal amphipathic helix are required for late endosome liposome penetration.** *A*, helical wheel plots of the N-terminal 17 amino acids of WT and mutant VP5 constructs (M1, M2, or M3). *Blue*, *white*, and *black circles* represent positively charged, hydrophobic, and glycine residues, respectively. *Filled black circle*, end of the sequence. Modified plots were generated from the Helical Wheel Web site. *B*, purified WT VP5 or mutants M2 and M3, lacking positive charged residues in their N-terminal amphipathic helix, were utilized in a pore formation assay as described in the legend to Fig. 2. Significant results are indicated (*, *p* < 0.01 compared with WT). *C*, effect of increasing amounts of NaCl on pore formation. Results from three independent repeats were normalized to liposomes treated or untreated with 0.1% Triton X-100. *Error bars*, S.D.

VP5 exhibits conserved positive charged residues in its two N-terminal amphipathic helices (amino acids 1–20) that have previously been shown to cause membrane disruption (35). These positive charges may interact with the anionic charge of PS or LBPA to allow initial membrane interaction in order to facilitate the penetration process. A recent atomic resolution structure of VP5 provides some structural evidence supporting this hypothesis. The structure indicates that this region of the N terminus forms a dagger-like domain that is hypothesized to be projected toward the host target membrane by an acid-induced conformational change (47). To interrogate this, VP5 mutants with substitutions of positive charged residues of its two helices to hydrophobic leucine residues were created because leucine displays the same branch length as lysine or arginine. We designed three sets of mutants (Fig. 3*A*): one substituting all positive charge in the first helix (mutant 1, K3L/R6L), one substituting positive charge in the second helix (mutant 2, R10L/K13L/K14L), and one substituting all positive charges in both helices (mutant 3, K3L/R6L/R10L/K13L/K14L). These mutants were expressed and purified as for WT VP5 (see “Experimental Procedures”); however, mutant 1 was poorly expressed (data not shown) and was not included in a pore formation assay. Mutants 2 and 3 yielded sufficient pure protein to test for pore formation and were shown to be trimeric structures following native PAGE analysis (data not shown). Both mutants displayed a significant reduction in LE liposome penetration with direct relation to positive charge content and penetration efficiency (Fig. 3*B*). The data indicate a role of ionic interactions between the N terminus of VP5 and target membranes in facilitating pore formation. As expected, these mutants also could not be recovered by reverse genetics in three separate attempts, suggesting an essential role of these positively charged residues in virus viability. Moreover, it was hypothesized that if VP5 is indeed interacting with LE liposomes via electrostatic forces, it would be possible to disrupt this process by increasing the ion content of the penetration reaction. Penetration assays were performed with LE liposomes and WT VP5 increasing the NaCl content of assay buffer. These results demonstrated that WT VP5 membrane penetration was inhibited by increasing the solvent dielectric of the assay medium (Fig. 3*C*), suggesting that electrostatic forces do indeed play a role in the process of VP5 membrane penetration. The solubility of VP5 was tested in each buffer condition by ultracentrifugation, which indicated no loss of VP5 solubility in all buffers tested (data not shown).

**FIGURE 4.**
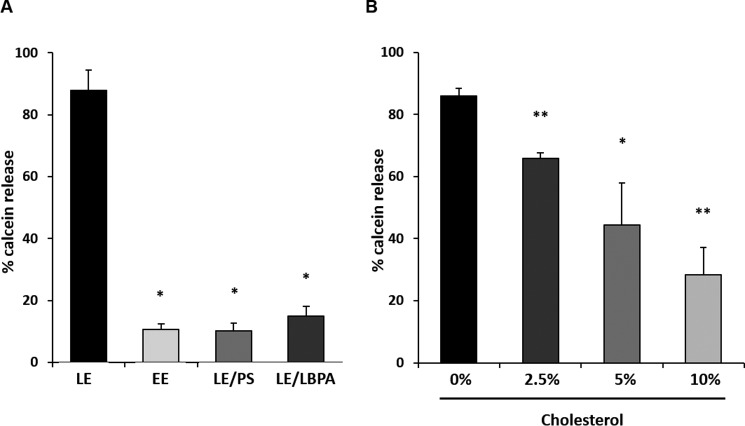
**VP5 pore formation requires LBPA of late endosome.** *A*, LE- or EE-mimicking liposomes were produced as before or had PS content swapped for LBPA (EE) or LBPA content swapped for PS (LE) as indicated and tested in a pore formation assay with purified VP5. Results were normalized to liposomes treated or untreated with 0.1% Triton X-100. Significant differences are indicated (*, *p* < 0.01 compared with LE 15% LBPA). *B*, LE liposomes were produced with the addition of increasing amounts of cholesterol and tested in a pore formation assay. Note that increasing cholesterol inhibits VP5 pore formation of LE liposomes. Results were normalized as in *A*. Experiments were performed in triplicate, and significant differences are indicated (*, *p* < 0.05; **, *p* < 0.01 compared with LE 15% LBPA). *Error bars*, S.D.

Given the role of ionic forces in VP5 penetration, we investigated whether PS could enable VP5 membrane penetration in the context of LE by swapping LBPA content with PS. Interestingly, the results demonstrated that despite possessing anionic charge, in the context of the more complex composition of LE membranes, PS was unable to support penetration by VP5 (Fig. 4*A*). This finding suggests that additional properties of LBPA, as well as negative charge, play a role in pore formation by VP5. LBPA has been demonstrated to increase membrane fluidity of constituent membranes, potentially due to lateral forces created by its conical profile (42). When LBPA was swapped for PS in EE-mimicking liposomes, no recovery of penetration was observed (Fig. 4*A*). The amount of PS in these membranes is only half that of LBPA in LE membranes, in addition to an increased sphingomyelin content. This increased sphingomyelin might have counteracted membrane fluidity of the lower concentration of LBPA in these liposome constituents. To test whether LE membrane penetration was affected by decreased membrane fluidity, LE liposomes were created with increasing cholesterol content. The results showed that increasing cholesterol with reduction in membrane fluidity do indeed decrease VP5 membrane penetration (Fig. 4*B*). Taken together, these data suggest that high membrane fluidity mediated by the physical properties of LBPA is necessary for VP5 membrane penetration.

##### Access to LBPA-enriched Membranes Is Required for Productive BTV-1 Entry in Cell Culture

Our *in vitro* assays demonstrated the necessity of LBPA in VP5 membrane penetration. To confirm this finding *in vivo*, we investigated whether productive BTV-1 entry required virion access to LBPA-enriched membranes in a cell culture model.

**FIGURE 5.**
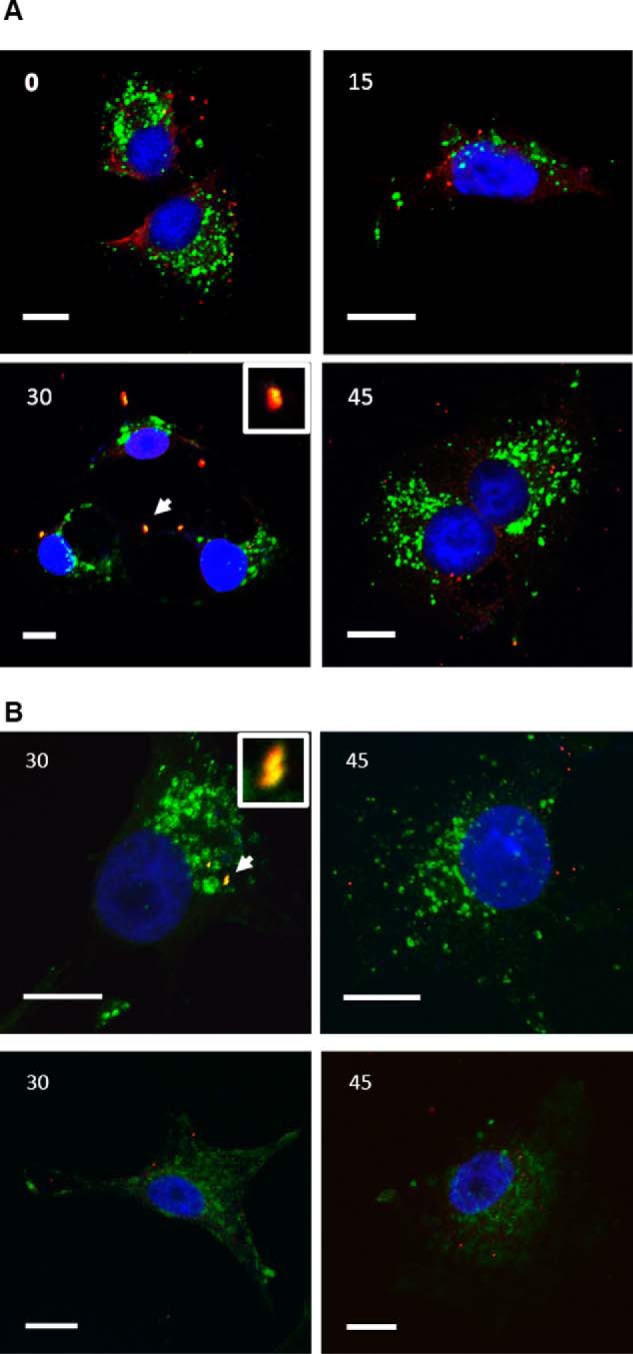
**Co-localization of VP5 and VP7 with LBPA in BTV-infected cells.** HeLa cells were fixed at 0, 15, 30, or 45 min postinfection. *A*, co-localization of VP5 (*red*) and LBPA (*green*). *B*, co-localization of VP7 (*red*) and LBPA (*green*, *top panels*) or Lamp1 (*green*, *bottom panels*). *Inset* in *30-min panels*, an *enlarged region* of co-localization indicated by a *white arrow. Bars*, 10 μm.

To assess whether VP5 could possibly interact with LBPA during virus entry, we determined whether VP5 and LBPA co-localized at 30 min postinfection (PI). LBPA is located in the LE (48), and it has been previously shown that BTV-1 co-localizes with the LE marker CD63 at 30 min PI (38). Cells were synchronously infected with an MOI of 10 and fixed and stained at time points 0, 15, 30, and 45 min PI, followed by double immunostaining of fixed HeLa cells. Results demonstrated that VP5 co-localizes with LBPA during cellular infection only at 30 min PI (Fig. 5*A*, *panel 30*), suggesting that the virus has the potential to interact with LBPA in cells during endocytosis. At 45 min PI, VP5 (Fig. 5*A*, *bottom right panel*) was no longer seen to co-localize with LBPA, indicating a migration out of LBPA-rich compartments.

Co-staining for LBPA and VP7 core protein was repeated at time points 30 and 45 min PI to detect the localization of the core (Fig. 5*B*, *top panels*). VP7 co-localized with LBPA at 30 min but to a lesser extent at 45 min PI, indicating that a viral core associated with VP5 was present within LBPA-rich compartments at 30 min PI. Subsequently, we examined whether the core (VP7) trafficked on to the lysosome by visualizing VP7 and Lamp1 co-localization at 30 and 45 min PI (Fig. 5*B*, *bottom panels*). Results showed a lack of substantial co-localization of Lamp1 with VP7 at both time points. This suggested that the majority of viral cores had migrated out of the endosomal trafficking pathway prior to reaching the lysosome.

U18666A is an amphipathic sterol that has been demonstrated to induce a Niemann-Pick C type disease phenotype within treated cells, by inhibiting cholesterol transport from lysosomal and late endosomal compartments (48, 49). Treatment of HeLa cells with 30 μm U18666A indicated an increase in cholesterol of LBPA-rich membranes (data not shown). To this effect, it was tested whether this U18666A-mediated increase in cholesterol of LBPA membranes inhibited BTV-1 entry into both PT and HeLa cells.

**FIGURE 6.**
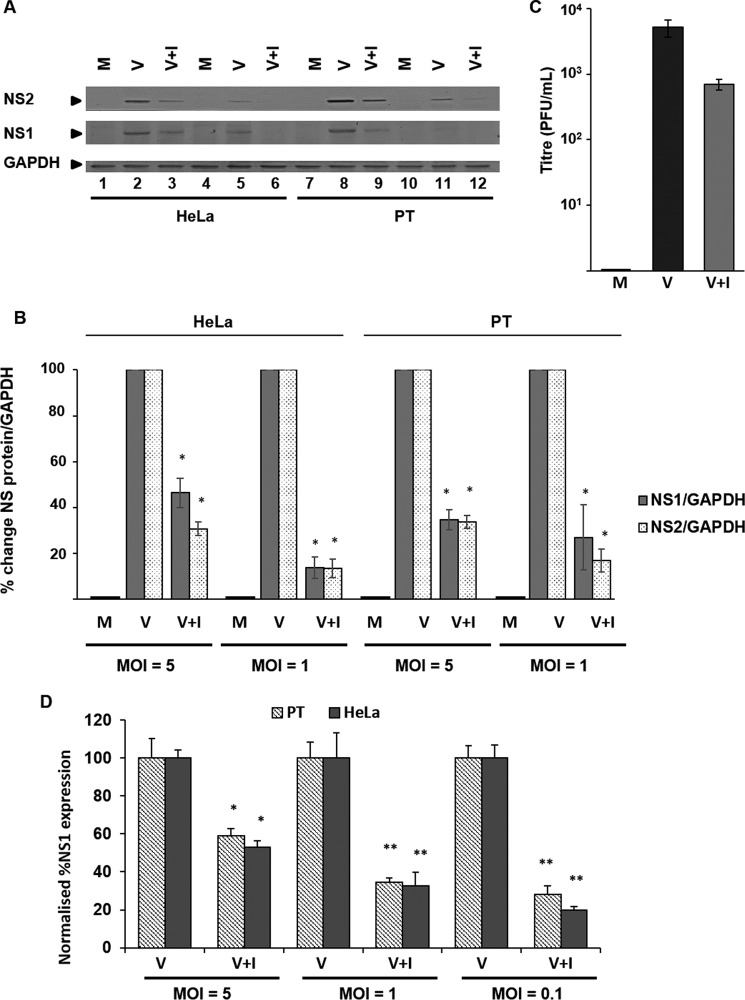
**Cholesterol accumulation in the late endosome inhibits BTV-1 infection.** HeLa and PT cells were treated with 30 μm of the late endosomal cholesterol transport inhibitor U18666A (+*I*) before infection with BTV-1. Uninfected cells (*M*) or cells infected but untreated with the drug (*V*) were used for comparison. *A*, non-structural proteins NS1 and NS2 were detected by Western blotting in cells infected at MOI = 5 (*lanes 1–3* and *7–9*) or MOI = 1 (*lanes 4–6* and *10–12*). *B*, densitometry analysis of the Western blotting expression data shown in *A. C*, quantification of virus titer by plaque assay (pfu/ml). Experiments were performed in quadruplicate, and *error bars* represent S.D. Significant results are indicated (*, *p* < 0.05). *D*, quantification of NS1 expression by immunoperoxidase assay. Results were normalized to NS1 expression from mock-treated infected and uninfected cells. Non-significant (*n.s.*; *p* > 0.05) and significant (*, *p* < 0.05; **, *p* < 0.01) results from three independent repeats are indicated. *Error bars*, S.D.

In both HeLa (Fig. 6*A*, *lanes 3* and *6*) and PT (Fig. 6*A*, *lanes 9* and *12*) cells, viral NS1 and NS2 protein levels were significantly decreased when compared with the untreated controls HeLa (Fig. 6*A*, *lanes 2* and *5*) and PT cells (Fig. 6*A*, *lanes 8* and *11*). Western blotting densitometry analysis showed that at an MOI of 5, NS1 protein levels decreased in HeLa and PT cells by ∼55 and ∼65%, respectively, and by ∼85 and ∼73% at an MOI of 1 (Fig. 6*B*). Similarly, at an MOI of 5, NS2 protein levels decreased in HeLa and PT cells by ∼70 and 66%, respectively, and by ∼87 and 83% at an MOI of 1 (Fig. 6*B*). Moreover, we also observed a ∼1 log_10_ decrease in virus titer in virus derived from previously treated cells (Fig. 6*C*). Furthermore, we carried out an immunoperoxidase-based assay, where we observed comparable decreases of NS1 (Fig. 6*D*), validating the assay for subsequent use. These data show that U18666A-mediated accumulation of cholesterol in LBPA-rich membranes was able to inhibit BTV-1 entry of both HeLa and PT cells.

**FIGURE 7.**
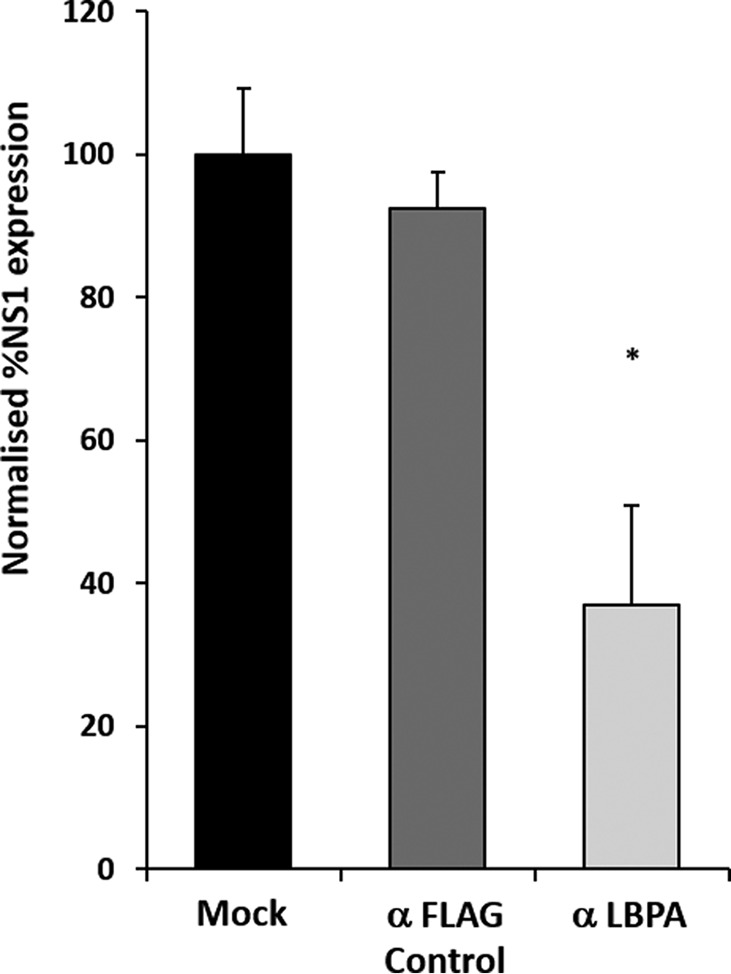
**Endocytosis of anti-LBPA antibody inhibits BTV-1 entry.** HeLa cells were treated with either anti-LBPA or anti-FLAG antibody before infection with BTV-1. The expression of NS1 was quantified by an immunoperoxidase assay. Results were normalized to NS1 expression in mock-infected cells. Significant (*, *p* < 0.05) results from three independent repeats are indicated. *Error bars*, S.D.

To support these findings, we used a commercially available anti-LBPA antibody, which has been shown to be endocytosed by cells in culture upon which it binds to LBPA in LE compartments. This binding disrupts the architecture of the LE (41) and has been shown to inhibit the entry of dengue virus, which also utilizes LBPA (50). In a titration experiment, no effect was seen in PT cells, potentially due to cell type-specific kinetics of antibody uptake. HeLa cells were incubated for 12 h in medium containing 50 μg ml^−1^ anti-LBPA or isotype-matched anti-FLAG antibody control or left untreated. After 12 h, medium was removed, and cells were washed and infected with BTV-1 at an MOI of 1. Cells were then fixed 12 h PI, and expression of NS1 protein was quantified by an immunoperoxidase assay. A reduction in NS1 expression was observed by preincubation with anti-LBPA antibody, supporting a specific role of this lipid in BTV-1 entry (Fig. 7). Taken together with *in vitro* observations, these results indicate that membrane fluidity plays a significant role in BTV-1 entry of cells.

**FIGURE 8.**
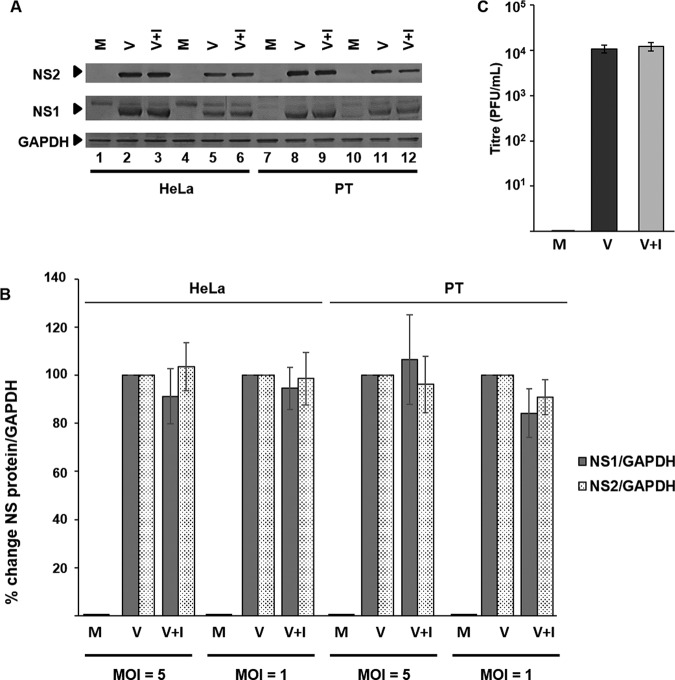
**Cholesterol accumulation in the late endosome subsequent to BTV-1 infection does not inhibit virus replication.** HeLa and PT cells were infected with BTV before treatment with 30 μm U18666A (+*I*). Uninfected cells (*M*) or cells infected but untreated with the drug (*V*) were used for comparison. *A*, expression of NS1 and NS2 proteins was detected by Western blotting in cells infected at MOI = 5 (*lanes 1–3* and *7–9*) or MOI = 1 (*lanes 4–6* and *10–12*). *B*, densitometry analysis of the Western blotting expression data from *A. C*, quantification of virus titer (PFU/ml). *Error bars*, S.D. values from four independent experiments. Significant difference from control is indicated (*, *p* < 0.05).

To exclude the possibility that the U18666A-mediated accumulation of cholesterol in LBPA-rich membranes could interfere with viral replication postentry, both HeLa and PT cells were treated with U18666A 4 h PI (Fig. 8*A*). Western blotting densitometry analyses revealed that regardless of MOI or cell line used, there was no significant decrease in either NS1 or NS2 protein levels, as had been observed during entry (Fig. 8*B*). Further, U18666A treatment did not affect virus titer (Fig. 8*C*).

##### LBPA May Facilitate VP5 Pore Expansion

**FIGURE 9.**
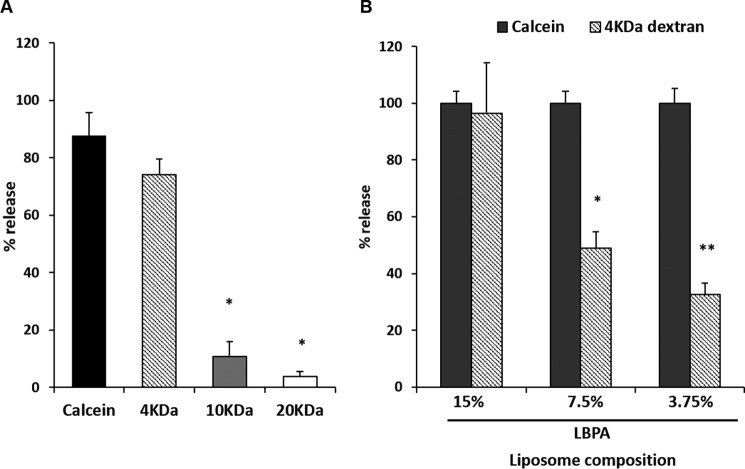
**VP5 forms pores of a discrete size in late endosome-mimicking liposomes.** *A*, late endosome-mimicking liposomes containing self-quenched concentrations of calcein or fluorescent dextrans of the indicated sizes (in kDa) were tested in a pore formation assay. Results were normalized to 0.1% Triton X-100-treated or -untreated liposomes. Significant differences are indicated (*, *p* < 0.01 compared with calcein release). *B*, late endosomes containing a decreasing amount of LBPA (swapped for PC) loaded with calcein or fluorescent 4-kDa dextran were tested in a pore formation assay. The release of 4-kDa dextran was normalized to that of calcein for liposomes of the same membrane composition. Significant results from three independent repeats are indicated (*, *p* < 0.05; **, *p* < 0.01). *Error bars*, S.D.

Based on our findings that VP5 penetrates LE-mimicking liposomes and that inhibition of access to LBPA membranes in cell culture inhibited BTV-1 entry, we next sought to analyze whether the pore formed in LE membranes could support core entry to the host cytosol. Because cell culture data supported the results derived from our *in vitro* assay system, it was deemed to be robust enough to draw conclusions about the nature of the pore formed by VP5 during cell entry. To this end, pore formation assays with LE liposomes and WT VP5 were performed, utilizing liposomes containing entrapped fluorescent dextrans of specific sizes. We utilized LE liposomes containing dextrans of 4, 10, and 20 kDa as well as the small molecule calcein as a positive control. In a VP5 penetration assay, LE liposomes were only capable of releasing calcein and 4-kDa dextran, suggesting that VP5 formed a discrete pore (Fig. 9*A*). The Stokes radii of these dextrans would place the diameter of the pore at >14 Å and <23 Å in size. Because there are 120 trimers of VP5 in each virion particle, this could provide sufficient local destabilization of the LE membrane to allow core egress. If calculated with the lowest assumption of 14-Å pore size diameter, the additive diameter of pores is 168 nm, sufficiently large for the diameter of the core, which is ∼70 nm; however, these trimers are not arranged linearly. It may be that concentrated local destabilization of the LE membrane is sufficient to facilitate the process of core egress.

Given the evidence that LBPA is key to the process of VP5 pore formation, we finally investigated whether this lipid has a role in modulating the pore formed by VP5. To address this, it was assessed whether lower LBPA concentration could affect the ability of 4-kDa dextran to be released from liposomes relative to the small molecule calcein. We performed a penetration assay utilizing LE liposomes loaded either with calcein or 4-kDa dextran, containing a titration of LBPA (Fig. 9*B*). For each membrane composition, the release of 4-kDa dextran was normalized to that of calcein for the same liposome composition. Interestingly, our results demonstrated that reducing the content of LBPA of LE liposomes decreased the relative release of 4-kDa dextran to calcein; this indirectly suggests, to some extent, that membrane LBPA content could allow for VP5 pore breathing or expansion.

## Discussion

Both enveloped and non-enveloped viruses must traverse the limiting membrane barrier in the host cell in order to initiate infection. This process demands interaction of the viral capsid with the host membrane that modifies the target membrane site. Enveloped viruses require fusion of their viral membrane with that of the host, a process that has been well characterized in both the viral protein components required (6, 51–54) and the host lipid factors that allow membrane fusion (55–57). In contrast to enveloped viruses, as yet, the lipid factors involved in the process of non-enveloped virus membrane remodeling/penetration remain poorly defined. The data presented here demonstrate a role of the host lipid factor LBPA in mediating host cell entry of the non-enveloped virus, BTV-1.

Liposome assays strongly suggest that LBPA potentiates pore formation by membrane penetration protein VP5 of BTV. The physical properties of this lipid that allow such action were delineated. Interrogation of physical factors of LBPA indicated a combined role of both anionic charge and membrane fluidic properties of this lipid. These properties appeared to be important to facilitate the penetration activity of VP5. PS liposome and mutant protein salt inhibition data suggest a model in which an ionic charge interaction between conserved positive charged residues of VP5 N-terminal amphipathic helix and anionic lipid may allow for initial membrane binding. However, this is necessary but not sufficient for pore formation in more complex liposome compositions, which more faithfully represent the makeup of cellular membranes encountered during virus endocytosis. It seems that a threshold level of membrane fluidity is also required for efficient VP5 pore formation, as indicated by LBPA and cholesterol titration.

Pharmacological inhibition in cell culture supports the *in vitro* model of membrane fluidity allowing BTV-1 entry and reliance on LBPA to facilitate this. U18666A-mediated cholesterol accumulation significantly decreases virus cell entry in both the model HeLa cell line and a more biologically relevant sheep-derived PT cell line. The exact effect of this inhibitor on BTV entry will require further studies because it is plausible that it may act to inhibit trafficking to the late endosomal compartment. Interestingly, U18666A has been shown to inhibit the entry of enveloped dengue virus, which has also been shown to rely on LBPA for cell entry from the late endosome (55, 58). For BTV-1, the effect of such inhibition was most pronounced at lower MOIs. Both enveloped and non-enveloped viruses have been shown to enter host cells through multiple pathways (59–62), the equilibrium of which may be shifted according to the amount of incoming virus utilizing each host cell pathway. It may be that at higher MOI, the major mode of host cell entry is shifted to a pathway that bypasses the late endosome as this pathway becomes saturated. BTV has been shown to enter cells via multiple pathways dependent on serotype. BTV-10 has been demonstrated to enter mainly via a clathrin-mediated endocytosis pathway (34), whereas BTV-1 has been implicated in utilizing a macropinocytosis mechanism (63). A more recent study with labeled virus using dynamin inhibition indicated that BTV-1 also utilizes clathrin-mediated endocytosis (38). It may be that the dynamic between these pathways is modulated by the MOI of infection of host cells. Macropincytosis delivers viral particles into macropinosomes, which traffic toward the lysosome, with which it fuses eventually (64, 65). The lysosomal membrane has also been shown to be enriched in LBPA (66), and as such, BTV may utilize this lipid at this site in the context of macropinocytic entry.

Along with BTV, other members of the Reoviridae also traffic through to the LE and lysosomes for cell entry. Mammalian orthoreovirus utilizes cathepsins of the late endosome to uncoat and expose viral μ1 protein for membrane penetration, which may occur in this compartment (67). However, the lipid components required to do so are not well characterized. Rotavirus has been shown to rely on the endosomal sorting complexes required for transport (ESCRT) machinery for host cell entry, and in addition, this process can be blocked by preincubating cells with an anti-LBPA antibody, strongly suggesting that it too requires access to LBPA for efficient membrane penetration (68). Given this, it seems that these complex non-enveloped viruses all require entry through the LE compartment. Our *in vitro* pore size data suggest a mechanistic reasoning to this observation, although more detailed studies will be required to confirm this initial observation. From our results, it appears that LBPA is able to allow the expansion of the pores produced from membrane penetration by VP5, and this occurred in a concentration-dependent manner of LBPA. It may be that the fluidic properties allow for breathing or expansion of the pores formed during penetration. Hypothetically, some viruses of the Reoviridae may require entry at the LE compartment because the LBPA content of these membranes allows for sufficient pore expansion upon penetration. This fluid expansion would enable more efficient delivery of a large core particle across the host cell membrane, thus initiating cell infection. This generalization requires further studies and may also provide a unique general therapeutic avenue for this virus family.

## Author Contributions

A. P., B. P. M., and P. R. designed the experiments, and A. P. and B.-P. M. conducted the experiments. P. R., A. P., and B. P.-M. analyzed the results and wrote the manuscript. P. R. secured funding and provided advice for the project.

## Footnotes

2 The abbreviations used are:

BTV: bluetongue virus
LE: late endosome
EE: early endosome
LBPA: 2,2′-dioleoyl lysobisphosphatidic acid
PC: phosphatidylcholine
PS: phosphatidylserine
MBP: maltose-binding protein
TEV: tobacco etch virus
MOI: multiplicity of infection
PI: postinfection.
